# C-CARE: comparing three years of anaphylaxis in children treated at the Montreal Children’s Hospital

**DOI:** 10.1186/1710-1492-10-S2-A3

**Published:** 2014-12-18

**Authors:** Sarah De Schryver, Elana Hochstadter, Ann Clarke, Sebastien LaVieille, Reza Alizadehfar, Alizee Dery, Christopher Mill, Harley Eisman, Moshe Ben-Shoshan

**Affiliations:** 1 Division of Allergy and Clinical Immunology, Department of Pediatrics, Montreal Children’s Hospital, McGill University Health Center, Montreal, QC, Canada; 2 Emergency Department, Department of Pediatrics, Montreal Children’s Hospital, McGill University Health Center, Montreal, QC, Canada; 3 Division of Rheumatology, Department of Medicine, University of Calgary, Calgary, AB, Canada; 4 Food Directorate, Health Canada, Ottawa, ON, Canada; 5 Department of Experimental Medicine, McGill University, Montreal, QC, Canada; 6 Department of Public Health, University of British Columbia, Vancouver, BC, Canada

## Background

The Cross-Canada Anaphylaxis Registry (C-CARE) aims to examine burden, triggers, management and temporal trends in anaphylaxis.

## Methods

Over a three-year period (April 2011 to April 2014), data were collected on anaphylaxis cases at the Montreal Children’s Hospital emergency department (ED). Cases were recruited either prospectively or identified retrospectively based on chart review through ICD10 codes related to anaphylaxis. Logistic regressions were conducted to determine the association between sociodemographic and clinical characteristics and development of severe reactions as well as epinephrine use.

## Results

Among 624 cases the median age was 5.7 years (IQR 2.4-11.7) and the majority (56.6%) were males. The percentage of anaphylaxis among all ED visits increased from 0.22% (95% CI 0.18%, 0.24%) in 2011 to 0.3% (95% CI 0.25%, 0.33%) in 2014, yielding a difference of 0.08% (95%CI, 0.03%, 0.13%). The major trigger was food (81.4%), mainly peanut and tree-nut. Most cases were moderate (70.2%) (breathing difficulties, stridor, diarrhea, crampy abdominal pain, recurrent vomiting).

Of all reactions 28.7% were not administered epinephrine. Almost 95% were prescribed or had an epinephrine auto-injector (71.6% Epipen , 22.7% Allerject).

Factors associated with severe reactions included history of peanut allergy, asthma and steroid treatment in ED. (Table 1)

**Table 1:** Logistic regressions assessing severe reactions and use of epinephrine

Severe reactions	
**Variable**	**OR, 95%CI**

Asthma	2.3 (1. 5)

Use of steroids in ED	2.5 (1.2, 5.2)

Peanut allergy	2.2 (1, 4.9)

**No use of epinephrine**	

Use of steroids in ED	0.2 (0.1, 0.4)

Known food allergy	0.3 (0.2, 0.5)

**Use of more than 2 epinephrine in ED**	

Use of epinephrine outside	0.06 (0.01, 0.6)

Use of steroids in ED	9.0 (2.6, 31)

Severe reaction	16.8 (4.7, 61)

Tree-nut allergy	5.4 (1.2, 24)

Management of anaphylaxis with at least one dose of epinephrine was associated with known food allergy and use of steroids in ED.

Administration of two or more epinephrine doses in ED was less likely in those who received epinephrine outside ED and more likely with severe reactions, reactions triggered by tree-nut and in those treated with steroids in ED.

## Conclusions

The percentage of anaphylaxis cases among all ED visits increased by almost 40% over a three-year period. Prompt use of epinephrine may prevent use of subsequent multiple doses of epinephrine in ED. Reactions triggered by tree-nut are more prone to require treatment with multiple doses of epinephrine.

